# Population characterization and parasitological assessment of the giant African snail ( *Achatina fulica*) in urban areas of Cartagena, Colombia

**DOI:** 10.12688/f1000research.28002.2

**Published:** 2021-03-22

**Authors:** Eder Cano-Pérez, Jaison Torres-Pacheco, Luis Barraza-Quiroz, Jorge Morelos-Muñoz, Doris Gómez-Camargo

**Affiliations:** 1 Grupo de Investigación UNIMOL, Facultad de Medicina, Universidad de Cartagena, Cartagena de Indias, Bolívar, 13001, Colombia; 2 Programa Ambiente y Salud, Departamento Administrativo Distrital de Salud (DADIS), Cartagena de Indias, Bolívar, 13001, Colombia; 3 Doctorado en Medicina Tropical, Facultad de Medicina, Universidad de Cartagena, Cartagena de Indias, Bolívar, 13001, Colombia

**Keywords:** Achatina fulica, biomass, population density, invasive species, plague, intermediary vector

## Abstract

**Background: **The giant African snail, *Achatina fulica*, is an invasive species recognized for being a serious agricultural pest and an intermediary vector for diverse parasites that cause diseases in humans. The knowledge of the state of African snail populations in urban areas is of great ecological and public health importance. Therefore, our objective was to characterize the status of giant African snail populations present in the city of Cartagena, Colombia, including the assessment of nematode parasites in the specimens.

**Methods.** Sites were visited following information from citizens affected by the presence of the African snail. The specimens were collected and transported to the laboratory; subsequently, they were weighed, measured, and classified by size. Dissections of lung tissue and soft organs were performed to search for and identify nematode parasites. Size measurement between the sampled sites was statistically compared and density and biomass indicators were established.

**Results**. In total, 204 snails were collected distributed among four sites within Cartagena city. Of these, 50% were juvenile specimens (10-40 mm). The size of the specimens showed significant differences between the sampling areas. The calculated density was between 0.0019-0.68 ind/m ^2^ and the biomass between 3.92-48.75 kg/ha. No presence of nematode parasites was observed in these specimens.

**Conclusions**. Densities and biomasses of *A. fulica* in Cartagena do not reach levels considered highly harmful. On the other hand, although no parasites were found in these snails, it is relevant to continue with studies on the human health risks that represent the presence of this invasive species in urban areas of Cartagena.

## Introduction

One of the greatest threats to global biodiversity, livelihoods, human and animal health, agriculture, and the local economy are invasive alien species ^1^. The giant African snail, *Achatina fulica*, (Bowdich 1822) is a gastropod native to Africa and considered one of the 100 worst invasive alien species in the world ^2, 3^. Currently, this species is widespread in tropical and subtropical areas on all continents due to the growth of international trade and increased transport that facilitate the introduction of this species, accidentally through agricultural or horticultural products or intentionally as a source of food or as a pet ^2, 4, 5^.

The giant African snail is recognized as an agricultural and garden pest ^5^. Its great reproductive capacity allows it to reach high densities and biomass in short times ^3, 5^, and its broad spectrum herbivorous diet allows it to consume more than 50 species of native and agricultural plants, all of which makes this species a serious pest with the ability to modify habitats and displace native species ^5^. The giant African snail is an intermediate host for various parasites, among which the nematodes *Angiostrongylus cantonensis* and *Angiostrongylus costaricensis* stand out, which cause eosinophilic meningoencephalitis and abdominal angiostrongyliasis in humans, respectively ^6, 7^. Recently, the presence of *A. cantonensis* in African snails in Colombian territory was confirmed, putting the population on alert due to the exposure of humans to the parasitic load of this snail, leading to the consideration of eosinophilic meningitis as a possible emerging disease in Colombia ^8^. Risk factors for infection in humans and pets by this and other nematodes include eating infected snails raw or undercooked, or food contaminated by snail slime or feces ^9, 10^.

In Colombia, the presence of *A. fulica* was registered for the first time in 2009 ^11^, since then the mollusk has been distributed in more than 20 departments, registering in all regions of the country ^12^. Particularly in the Colombian Caribbean Region, the growth of *A. fulica* populations has been documented in the department of Sucre ^12– 14^. In Cartagena, Bolívar, journalistic media reported the presence of the giant African snail south of the city in 2016. However, since its appearance, there has been no scientifically documented record of the presence and distribution of this species in urban areas of the city, as well as its impact on human health. Therefore, the objective of this study was to generate the baseline of reference information on the African snail populations present in the Cartagena city, including the assessment of nematode parasites in the specimens.

## Methods

### Study area

Cartagena (10 ° 23'59 '' N 75 ° 30'52 '' W), capital of the department of Bolívar located within the Colombian Caribbean region, is a city of low ecotope or coastal, presenting altitudes between 1 and 9 meters above sea level, a temperature average of 27.5°C, a relative humidity of 80% and an annual rainfall of 870 mm.

### Collection of specimens

The search for, and collection of, the snails was carried out between July and October 2019, a period that included the rainy season in the region and which the proliferation of the giant African snail is favored. The sampling consisted of visiting the zones within the neighborhoods of the city Cartagena previously informed by citizens affected by the presence of the snail in their homes and surroundings. At each place, a 50-minute sampling effort was made by two people in a single visit. During the inspections, searches were carried out in gardens, under undergrowth and rubble, or between crevices where they could take refuge. The specimens were collected manually using gloves and face masks and transported in easy-sealing bags to the laboratory of the UNIMOL research group in the University of Cartagena. As this was a monitoring study, all the snails available during collections were included for analyzes, no exclusion criteria were applied for the collection of the snails.

### Morphometric analysis

The collection included live individuals of all sizes, which were measured using a digital caliper (0.01 mm) and weighed in a weighing machine (1 gr). The length of the shell was used as a descriptor of the total size of each individual, so size classes were defined as the following: class 1, to which newly hatched individuals and up to 10 mm were assigned; class 2, juveniles (10–40 mm); class 3, young adults (40–70 mm) and class 4, adults (> 70 mm) ^15^.

### Parasitological analysis

Specimens larger than 50 mm have a greater probability of carrying nematode larvae ^16^, even so, we decided to evaluate specimens greater than 30 mm to increase the probability of findings. The snails were washed externally and were put to sleep by thermal shock by introducing them in ice water, once they were numb, the shell was broken by using dissection scissors to extract the lung tissue and the rest of the soft organs. The search and collection methods for nematode parasites described by Córdoba *et al*. ^17^ were used. Briefly, the lung tissue was cut in 1 × 1 cm pieces and placed on microscope slides for observation under a stereoscope. Likewise, the other organs were cut and examined by compression between two glass pieces.

### Data analysis

As data were not normally distributed, the snail size measurements between the sites were compared using the Kruskal-Wallis and Bonferroni tests post hoc, using the statistical software SPSS v.19.0 (IBM Corp, Armonk, NY) with a statistic significance defined as p<0.05. Also, the population density of each zone was calculated according to the number of individuals collected per effective area sampled (Individuals/m ^2^) and the biomass per unit area (kg/hectares) was established. The google earth Pro 7.3.3.7786 polygon was used to determine the sampled area of each zone.

### Ethical aspects

Giant African snail has been declared in Colombia as an invasive species, for which legal research permits are not required. This study was adjusted to the management regulations for *A. fulica* issued by the Ministry of Environment, 2011 ^18^. Also, during the execution of the study, efforts were made to reduce animal suffering during sacrifices, for this, the snails were put to sleep in ice water until their death before the dissections.

## Results

**Figure 1.**
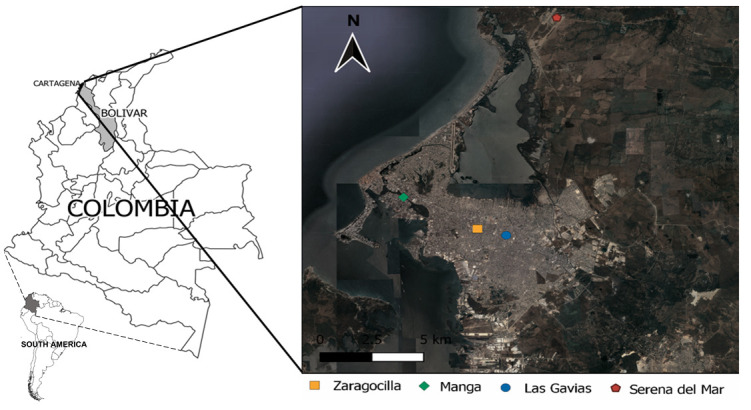
Sites where snails were found in Cartagena, Colombia.

During the period studied 204 snails were collected, distributed in four zones of four Cartagena neighborhoods, for which the presence of *A. fulica* was indicated by the community ( Figure 1). The inspected sites where the snails were collected corresponded principally to extensive gardens and other spaces inside of private properties and residential condominiums (Manga, Las Gavias, and Serena del Mar). At these sites the snails were found in different substrates, such as plants, tree roots, grasses, under litter, flowerpots, and ornamental plants; In Zaragocilla, snails were found aggregated in rubble inside an educational institute.

Overall, 50% of the total material collected corresponded to class 2 snails, followed by class 3 (42.64%) and class 4 (7.35%). In particular, in the Zaragocilla and Las Gavias sites, class 3 individuals predominated with 44.11% and 52.33%, respectively, in Manga, class 2 specimens were more abundant with 73.33% ( Table 1).

**Table 1.** Size class distribution of *A. fulica* in Cartagena, Colombia.

Site	Class 1	Class 2	Class 3	Class 4	Total
**Zaragocilla**	0	10	15	9	34
**Manga**	0	44	15	1	60
**Las Gavias**	0	48	56	3	107
**Serena del Mar**	0	0	1	2	3
**Total**	0	102	87	15	204

Class 1: newly hatched individuals (up to 10 mm); class 2: juveniles (10 to 40 mm); class 3: young adults (40 to 70 mm); class 4: adults (> 70 mm); total: total of snails collected

Mean size and weight per site is shown in Table 2. The Kruskal-Wallis test showed a significant difference between the sizes of the most abundant African snail population groups (Zaragocilla, Manga, and Las Gavias sites) (p<0.01). Likewise, Bonferroni-adjusted pairwise comparisons indicated that differences existed between each site (p<0.01 between each pairing). The population density established in each point, as well as the estimated biomass according to the calculated total weight, is presented in Table 3.

**Table 2.** Analysis morphometric the population groups of *A. fulica* in Cartagena, Colombia.

Parameters	Site	n	Mean	Range	SD
**Size** **(mm)**	Zaragocilla	34	57.2	24.3–87.9	17.99
	Manga	60	36.2	20.5–78	11.44
	Las Gavias	107	42.6	28.3–80	8.08
	Serena del Mar	3	69	59.4–77.6	9.15
**Weight** **(g)**	Zaragocilla	34	26.4	3–86	20.8
	Manga	60	7.1	1–25	5.56
	Las Gavias	107	9,7	3–38	5.36
	Serena del Mar	3	21	17–25	4

n: number of snails collected, SD: standard deviation.

**Table 3.** Density and biomass calculated in the population groups of *A. fulica* in Cartagena, Colombia.

Site	n	Sampled area (m ^2^)	Density (ind/m ^2^)	Total weight (g)	Biomass (kg/ha)
**Zaragocilla**	34	747	0.0455	898	12.02
**Manga**	60	88	0.6818	429	48.75
**Las Gavias**	107	2443	0.0438	1048	4.28
**Serena del Mar**	3	1605	0.0019	63	3.92

n: number of snails collected.

Regarding the parasitological analyzes, all the observed lungs were found healthy (free of cysts), likewise, no free larvae were observed in the slime or the rest of the soft organs, showing that these specimens were not infected with nematode parasites according to the methodology used.

## Discussion

Understanding the populations of giant African snail present in different areas of Colombia is of great ecological, agricultural, and public health importance. The current study constitutes the first scientifically documented record of the presence of the species *A. fulica* in urban areas of the city of Cartagena.

It is known that the African snail can reach sizes of up to 300 mm in the length of the shell ^19^. In contrast, other studies establish that the size naturally reached by *A. fulica* is around 100 mm ^20, 21^. Even so, the mean values reported are usually much smaller and vary between 30 and 60 mm ^13, 14, 22– 25^. Comparatively, our results from Cartagena agree with these last values, obtaining average sizes between 36.2–69 mm. Patterns in snail size can reflect the age structure of the population in a given area. It has been proposed that the establishment of *A. fulica* in new areas takes place in three stages: exponential, known as a long phase with vigorous individuals; stable, with a prevalence of a variable shell size among young and adult, and the decline phase, where young individuals are prevalent ^22^. This suggests that the populations obtained from *A. fulica* collected in Cartagena are in the stable phase, presenting a variety of young and adult individuals.

The population density of *A. fulica* reported in this study was between 0.0019–0.6818 ind/m ^2^, these density values are similar to those obtained by De la Ossa *et al*. in studies carried out with snails collected in urban areas of the city of Sincelejo and other municipalities of the department of Sucre, these authors reported densities in a range of 0.0031–0.205 ind/m ^2^ ^13, 14, 24^. Other similar values were reported in a study performed in Ilha Porchat, Brazil, obtaining a density of 0.07 ind/m ^2^ ^3^. Investigations such as those carried out in Havana, Cuba, showed a considerably lower density of snails (0.00015 ind/m ^2^) ^26^. In contrast, densities of 1.1–4.6 ind/m ^2^ have been reported in different departments of Colombia ^27^, 0.06–8 ind/m ^2^ in Northeast Brazil ^28^, and an average of 8.4 ind/m ^2^ in Puyo, Ecuador ^22^. On the other hand, studies performed in Puerto Iguazú y Corrientes, Argentina, recorded a much higher average density, which reaches 107.6 ind/m ^2^ and 118.6 ind/m ^2^, respectively ^15, 29^. It has been mentioned that the areas highly affected by the giant African snail present densities of 10 ind/m ^2^ or more ^12, 30^; this similarly occurs with regards to biomass, where devastating values of up to 780 kg/ha are estimated for areas which are highly affected ^12, 31^. If that is the case, the densities and biomasses obtained in the snail populations of Cartagena are not very harmful.

Nematodes such as *A. cantonensis* and *A. costaricensis* cause eosinophilic meningoencephalitis and abdominal angiostrongyliasis, respectively, in humans. The life cycle of these nematodes involves rodents as the definitive host and mollusks of various species, including *A. fulica*, as intermediate hosts ^7^. The snails examined in this work did not show evidence of infection by nematode parasites, one of the reasons could be the environment in which these snails were found. It has been noted that the African snail prefers sites such as garbage dumps, landfills, and empty lots, places where there is a greater probability of contact with rats, necessary for the nematodes to complete their life cycle ^17^. In our case, more than 80% of the analyzed snails were collected in gardens of remarkably well-kept properties given the exclusivity of the sites, and according to interviews with various neighbors, rodents do not frequent these spaces.

In conclusion, although the densities of *A. fulica* estimated do not reach values considered highly harmful, the presence of this invasive species in properties and residential areas of Cartagena is worrying. Most of the snails were found associated with gardens, which suggests that the introduction and dispersal of this species within the city are related to the transport of organic fertilizers or other garden products, which could convert this species into a serious pest. Similarly, although no parasites were found in these snails, it is necessary to continue carrying out studies on the effect of this species on human health, as well as training campaigns on the management and control of the giant African snail in the city.

## Data availability

Figshare: Raw data file_African Snail Project.csv, https://doi.org/10.6084/m9.figshare.13288808 ^32^.

This project contains the following underlying data:

- Raw data file_African Snail Project.csv (original raw data)

Data are available under the terms of the Creative Commons Attribution 4.0 International license (CC-BY 4.0).
