# Supplementary material for: Population characterization and parasitological assessment of the giant African snail ( Achatina fulica) in urban areas of Cartagena, Colombia
Source: F1000Res. 2021 Mar 22;10:77. Originally published 2021 Feb 5. [Version 2] doi: 10.12688/f1000research.28002.2 (PMC8280942; doi:10.12688/f1000research.28002.2)
Supplement: Peer review history

# Peer review: Reviewer response for version 2

**DOI:** 10.5256/f1000research.55535.r86470

**Published:** 2021-07-14

**Authors:** Roshmi Rekha Sarma

**Affiliations:** 1 Evolution and Ecology Research Centre, School of Biological, Earth and Environmental Sciences, University of New South Wales, Sydney, NSW, Australia

In the article “Population characterization and parasitological assessment of the giant African snail ( *Achatina fulica*) in urban areas of Cartagena, Columbia, Eder Cano-Perez and co-authors attempt to identify Giant African snail (GAS) population in the city Cartagena, Columbia and investigate the presence of nematode parasites in the collected specimens. Giant African snails are a highly invasive species and if remained unchecked can affect agriculture, native biodiversity, human health, and the economy. The authors in this paper provide an initial status of this invasive species since its introducing in 2016, whether they are carriers of nematode parasites and if they pose any potential human health risk. This article addresses the need for early assessment and collecting distribution information of invasive species to prepare and make better management plans. Despite the small Spatio-temporal distribution of the study, it makes an important contribution to invasive species distribution and its potential environmental and health risks.

 The authors have collected specimens and location information for 204 snails from four zones within the city of Cartagena. They recorded their body weight, shell size, snail density per area, presence-absence of parasites within organs of each specimen. The authors found that 50% of the snails were juveniles (10-40 mm) followed by adults (40-70 mm and >70 mm). They also found a significant differences in sizes across the different zones. Interestingly the specimens collected were healthy and free from any cysts or larvae. The authors have clearly cited the current literature surrounding the invasion of giant African snails, especially across South America. The study design, sample collection and analysis as well as limitations of the collection sites are also well explained and justified. However, the raw data presented seems to be incomplete as a result the use and interpretation of the statistical models and analysis do not ensure full reproducibility of the data.

 Here are some queries suggestions:

1. The authors indicate the number of samples collected is 204 but the raw data file lists only 50 individuals. As a result, I was not able to analyze the data myself. Additionally, can they provide details of how the variables were analyzed in the model?
2. It would perhaps be helpful if the authors provided information (in form of the column data) of the calculated population density and biomass per unit area in the raw data file. 
3. Can the authors clarify why they decided not to normalize the data? The weight data can be ln-transformed, and the shell length can normalize using sqrt function.
4. The column “number of snails collected per site” is missing or if it is in the data file, the column title is missing.
5. Can the authors please elaborate on the column shell length? What do the two values indicate and some rows do not have two values.
6. Graphical representation of the statistical analysis will help in understanding the paper more as it will indicate the significant differences between the population density across the different collection sites.
7. The authors mention the Giant African snail affect the citizens of Cartagena; it would be helpful if they can shed light on what those effects are? For example, they are in general a nuisance or their presence or the sight of the snails.
8. Under the section “Morphometric analysis” it can be useful if the authors provide information as standard errors as “+/-” for digital callipers and weighing machine.
9. I would like to see some more discussion about the different phases of the *A.fulica* establishment and its potential impact (if any) in the future in Cartagena.
10.  I would like to see some more discussion on the population density of the snails in Cartegena and what it can possibly imply for the future.

Is the work clearly and accurately presented and does it cite the current literature?

Yes

If applicable, is the statistical analysis and its interpretation appropriate?

Partly

Are all the source data underlying the results available to ensure full reproducibility?

No

Is the study design appropriate and is the work technically sound?

Yes

Are the conclusions drawn adequately supported by the results?

Partly

Are sufficient details of methods and analysis provided to allow replication by others?

Partly

Reviewer Expertise:

Invasive species, Data analysis

I confirm that I have read this submission and believe that I have an appropriate level of expertise to confirm that it is of an acceptable scientific standard, however I have significant reservations, as outlined above.

# Peer review: Reviewer response for version 2

**DOI:** 10.5256/f1000research.55535.r87814

**Published:** 2021-07-12

**Authors:** Jenny J Chaparro Gutiérrez

**Affiliations:** 1 CIBAV Research Group, Veterinary Medicine School, Faculty of Agrarian Sciences, University of Antioquia, Antioquia, Colombia

The study of the African giant snail, *Lissachatina fulica*'s distribution and possible source of zoonotic agents is critical. This study describes the pest's distribution in Cartagena, and its findings are valuable as primary data in the area.

 ​​​​​​Several parts of the manuscript, however, need to be supplemented or clarified:

 1.The snail's genus and species are *Lissachatina fulica*.

 2. It is unclear how they determined the sample size or the number of calls they would answer to define sample size.

 3. Although the dissection method was chosen to search for nematodes in snails, the limitations of this methodology in comparison to enzymatic digestion methods were not mentioned. It is critical to include this when discussing the effects of parasite absence.

 4.The cited literature is incomplete; it does not include all reports of studies on the presence of the snail in Colombia, and studies with relevant findings of nematodes with or without pathogenic potential in animals are omitted e.g:

- Guerrero *et al. *(2018) ^1^
- Penagos-Tabares *et al.* (2019) ^2^
- Heredia *et al.* (2018) ^3^
- Lange et al. (2018) ^4^

5.Interviews with neighbours are mentioned in the discussion, but this is not reflected in the materials and methods, nor in the information analysis. However, no mention is made of the community's reaction to the presence of the snail. There is mention of a potential impact on public health, but no mention of consumption habits or home gardens with the data of interviews. Please clarify.

 6.The conclusion emphasizes the significance of continuing to conduct studies and management and control campaigns; I propose that it be stated whether they exist in Colombia or should be designed, and whether they should be articulated with the ICA or the National Ministry of Health.

Is the work clearly and accurately presented and does it cite the current literature?

Partly

If applicable, is the statistical analysis and its interpretation appropriate?

Yes

Are all the source data underlying the results available to ensure full reproducibility?

Yes

Is the study design appropriate and is the work technically sound?

Partly

Are the conclusions drawn adequately supported by the results?

Partly

Are sufficient details of methods and analysis provided to allow replication by others?

Partly

Reviewer Expertise:

Veterinary Parasitology

I confirm that I have read this submission and believe that I have an appropriate level of expertise to confirm that it is of an acceptable scientific standard, however I have significant reservations, as outlined above.

## References

[1] : Achatina fulica (Mollusca: Achatinidae) Naturally Infected with Caenorhabditis briggsae (Dougherty and Nigon, 1949) (Nematoda: Rhabditidae). *J Parasitol* .104(6) : 10.1645/15-807 679-684 10.1645/15-807 30240329

[2] : The invasive giant African snail Lissachatina fulica as natural intermediate host of Aelurostrongylus abstrusus, Angiostrongylus vasorum, Troglostrongylus brevior, and Crenosoma vulpis in Colombia. *PLoS Negl Trop Dis* .13(4) : 10.1371/journal.pntd.0007277 e0007277 10.1371/journal.pntd.0007277 31002674PMC6493767

[3] : In vitro culture of L3 larvae of nematodes obtained from the African giant snail Lissachatina fulica (Mollusca: Gastropoda) in Santa Fe de Antioquia. *Biomedica* .2018;38(0) : 10.7705/biomedica.v38i3.3408 24-29 10.7705/biomedica.v38i3.3408 30184375

[4] : Regional report on Angiostrongylus vasorum in Colombia: Genetic similarity to European lineage. *Vet Parasitol Reg Stud Reports* .13: 10.1016/j.vprsr.2018.03.004 21-23 10.1016/j.vprsr.2018.03.004 31014876

# Peer review: Reviewer response for version 2

**DOI:** 10.5256/f1000research.55535.r81812

**Published:** 2021-04-20

**Authors:** Diego E. Gutiérrez Gregoric

**Affiliations:** 1 División Zoología Invertebrados, Museo de La Plata, Buenos Aires, Argentina; 2 CONICET, Buenos Aires, Argentina

Responses to comments have been satisfactory.

Is the work clearly and accurately presented and does it cite the current literature?

Yes

If applicable, is the statistical analysis and its interpretation appropriate?

Partly

Are all the source data underlying the results available to ensure full reproducibility?

Yes

Is the study design appropriate and is the work technically sound?

Yes

Are the conclusions drawn adequately supported by the results?

Yes

Are sufficient details of methods and analysis provided to allow replication by others?

Partly

Reviewer Expertise:

Malacology

I confirm that I have read this submission and believe that I have an appropriate level of expertise to confirm that it is of an acceptable scientific standard.

# Peer review: Reviewer response for version 1

**DOI:** 10.5256/f1000research.30971.r79127

**Published:** 2021-03-05

**Authors:** Diego E. Gutiérrez Gregoric

**Affiliations:** 1 División Zoología Invertebrados, Museo de La Plata, Buenos Aires, Argentina; 2 CONICET, Buenos Aires, Argentina

The present study focuses on an invasive alien species with potential effects on human health and agriculture. The population and parasitological analysis in urban areas of Cartagena, Colombia is highlighted, in such a way as to evaluate the role of these snails as potential transmitters of parasites. As the authors postulate, these studies must be constant, since in Colombia itself parasites have already been registered in this snail.

 With regard to data collection, the estimate of density is not clear, nor is the number of samplings. Did they go to the same site more than once? Is a site the same as a house? These points should be better clarified in the methodology and in the results tables.

**Minor Comments**

- Keywords: *Achatina fulica* must go in italic.
- Introduction: The authors mention the first citation of *Achatina fulica *in Colombia for 2010, however in 2009 it had already been cited by Borrero et al. (2009, Tentacle N ° 17).
- In Table 1 you must indicate if it is the total number of individuals collected.
- For further parasitological studies on snails, the authors should use other techniques for the observation of larvae, in addition to those used here (see Valente *et al* 2017, Veterinary Parasitology).

Is the work clearly and accurately presented and does it cite the current literature?

Yes

If applicable, is the statistical analysis and its interpretation appropriate?

Partly

Are all the source data underlying the results available to ensure full reproducibility?

Yes

Is the study design appropriate and is the work technically sound?

Yes

Are the conclusions drawn adequately supported by the results?

Yes

Are sufficient details of methods and analysis provided to allow replication by others?

Partly

Reviewer Expertise:

Malacology

I confirm that I have read this submission and believe that I have an appropriate level of expertise to confirm that it is of an acceptable scientific standard, however I have significant reservations, as outlined above.

# Response

**Published:** 2021-03-16

**Authors:** Eder Cano Pérez

**Affiliations:** University of Cartagena, Colombia

Dear reviewer,

 Thank you for the comments and suggestions to our manuscript.
